# Hearing Loss Prevalence, Years Lived With Disability, and Hearing Aid Use in the United States From 1990 to 2019: Findings From the Global Burden of Disease Study

**DOI:** 10.1097/AUD.0000000000001420

**Published:** 2023-09-15

**Authors:** Lydia M. Haile, Aislyn U. Orji, Kelly M. Reavis, Paul Svitil Briant, Katia M. Lucas, Fares Alahdab, Till Winfried Bärnighausen, Arielle Wilder Bell, Chao Cao, Xiaochen Dai, Simon I. Hay, Golnaz Heidari, Ibraheem M. Karaye, Ted R. Miller, Ali H. Mokdad, Ebrahim Mostafavi, Zuhair S. Natto, Shrikant Pawar, Juwel Rana, Allen Seylani, Jasvinder A. Singh, Jingkai Wei, Lin Yang, Kanyin Liane Ong, Jaimie D. Steinmetz

**Affiliations:** 1 Institute for Health Metrics and Evaluation, University of Washington, Seattle, WA, USA; 2 National Center for Rehabilitative Auditory Research, US Department of Veterans Affairs—Portland Healthcare System, Portland, OR, USA; 3 Mayo Evidence-based Practice Center, Mayo Clinic Foundation for Medical Education and Research, Rochester, MN, USA; 4 Heidelberg Institute of Global Health (HIGH), Heidelberg University, Heidelberg, Germany; 5 T.H. Chan School of Public Health, Harvard University, Boston, MA, USA; 6 Department of Global Health and Social Medicine, Harvard University, Boston, MA, USA; 7 Department of Social Services, Tufts Medical Center, Boston, MA, USA; 8 Program in Physical Therapy, Washington University in St. Louis, St. Louis, MO, USA; 9 Department of Health Metrics Sciences, School of Medicine, University of Washington, Seattle, WA, USA; 10 Independent Consultant, Santa Clara, CA, USA; 11 School of Health Professions and Human Services, Hofstra University, Hempstead, NY, USA; 12 Pacific Institute for Research & Evaluation, Calverton, MD, USA; 13 School of Public Health, Curtin University, Perth, WA, Australia; 14 Department of Medicine, Stanford University, Palo Alto, CA, USA; 15 Stanford Cardiovascular Institute, Stanford University, Palo Alto, CA, USA; 16 Department of Dental Public Health, King Abdulaziz University, Jeddah, Saudi Arabia; 17 Department of Oral Health Policy and Epidemiology, Harvard University, Boston, USA; 18 Department of Genetics, Yale University, New Haven, CT, USA; 19 Department of Epidemiology, Biostatistics and Occupational Health, McGill University, Montreal, QC, Canada; 20 Research and Innovation Division, South Asian Institute for Social Transformation (SAIST), Dhaka, Bangladesh; 21 National Heart, Lung, and Blood Institute, National Institute of Health, Rockville, MD, USA; 22 School of Medicine, University of Alabama at Birmingham, Birmingham, AL, USA; 23 Medicine Service, US Department of Veterans Affairs (VA), Birmingham, AL, USA; 24 Department of Epidemiology and Biostatistics, University of South Carolina, Columbia, SC, USA; 25 Cancer Epidemiology and Prevention Research, Alberta Health Services, Calgary, BC, Canada; 26 Department of Oncology, University of Calgary, Calgary, AB, Canada.

**Keywords:** Hearing loss, Epidemiology, Tinnitus, Hearing aids, Hearing healthcare

## Abstract

**Objectives:**

This article describes key data sources and methods used to estimate hearing loss in the United States, in the Global Burden of Disease study. Then, trends in hearing loss are described for 2019, including temporal trends from 1990 to 2019, changing prevalence over age, severity patterns, and utilization of hearing aids.

**Design:**

We utilized population-representative surveys from the United States to estimate hearing loss prevalence for the Global Burden of Disease study. A key input data source in modeled estimates are the National Health and Nutrition Examination Surveys (NHANES), years 1988 to 2010. We ran hierarchical severity-specific models to estimate hearing loss prevalence. We then scaled severity-specific models to sum to total hearing impairment prevalence, adjusted estimates for hearing aid coverage, and split estimates by etiology and tinnitus status. We computed years lived with disability (YLDs), which quantifies the amount of health loss associated with a condition depending on severity and creates a common metric to compare the burden of disparate diseases. This was done by multiplying the prevalence of severity-specific hearing loss by corresponding disability weights, with additional weighting for tinnitus comorbidity.

**Results:**

An estimated 72.88 million (95% uncertainty interval (UI) 68.53 to 77.30) people in the United States had hearing loss in 2019, accounting for 22.2% (20.9 to 23.6) of the total population. Hearing loss was responsible for 2.24 million (1.56 to 3.11) YLDs (3.6% (2.8 to 4.7) of total US YLDs). Age-standardized prevalence was higher in males (17.7% [16.7 to 18.8]) compared with females (11.9%, [11.2 to 12.5]). While most cases of hearing loss were mild (64.3%, 95% UI 61.0 to 67.6), disability was concentrated in cases that were moderate or more severe. The all-age prevalence of hearing loss in the United States was 28.1% (25.7 to 30.8) higher in 2019 than in 1990, despite stable age-standardized prevalence. An estimated 9.7% (8.6 to 11.0) of individuals with mild to profound hearing loss utilized a hearing aid, while 32.5% (31.9 to 33.2) of individuals with hearing loss experienced tinnitus. Occupational noise exposure was responsible for 11.2% (10.2 to 12.4) of hearing loss YLDs.

**Conclusions:**

Results indicate large burden of hearing loss in the United States, with an estimated 1 in 5 people experiencing this condition. While many cases of hearing loss in the United States were mild, growing prevalence, low usage of hearing aids, and aging populations indicate the rising impact of this condition in future years and the increasing importance of domestic access to hearing healthcare services. Large-scale audiometric surveys such as NHANES are needed to regularly assess hearing loss burden and access to healthcare, improving our understanding of who is impacted by hearing loss and what groups are most amenable to intervention.

## INTRODUCTION

The Institute for Health Metrics and Evaluation (IHME) produces results for the GBD study, the largest and most comprehensive attempt to quantify disease epidemiology worldwide (“Hearing Loss Prevalence and Years Lived with Disability, 1990–2019: Findings from the Global Burden of Disease Study 2019—The Lancet” 2021). The most recent iteration of this study was published in October 2019, providing updated estimates for 369 diseases and injuries (Vos et al. 2020). GBD 2019 estimated hearing loss burden for 204 countries and territories between 1990 and 2019 by sex, age, etiology, and severity.

Hearing loss is linked to a reduction in several quality of life measures, including social wellbeing (Bott & Saunders 2021), cognition (Lin 2011), and dementia (Livingston et al. 2020). Hearing loss additionally poses considerable stress upon health systems, with estimates suggesting that hearing loss produces over $3 billion in excess medical expenditures in the United States alone (National Academies of Sciences 2016). Tinnitus, defined as the perception of sound in the absence of acoustic stimuli, is often concurrent with hearing loss and has adverse effects on concentration and sleep patterns, with prevalence estimates ranging from 8% to 20% in the US population (Henry et al. 2005; Bhatt et al. 2016).

Modifiable risk factors for hearing loss and tinnitus such as occupational and recreational noise exposure underscore the need for preventive strategies to reduce burden (Carroll et al. 2017). Although the disability posed by hearing loss can be mitigated through assistive devices, technologies, and accessible speech-language education, hearing healthcare coverage varies by U.S. state, and impacted individuals commonly incur significant out-of-pocket costs (Arnold et al. 2017). Similarly, research suggests that many tinnitus patients are not offered treatment aligned with clinical protocol (Bhatt et al. 2016). Researchers and providers have called for policy action to increase the affordability and accessibility of hearing healthcare through transparent fee structures, central standards and regulation for hearing technologies, and promotion of hearing healthcare in medical visits (National Academies of Sciences 2016). The large burden of hearing loss and tinnitus coupled with a lack of access to preventive and healthcare-based services presents an important problem within the American health system.

While several large-scale surveys (“NHANES Survey Methods and Analytic Guidelines”) have been conducted in the United States assessing hearing loss, tinnitus, and hearing aid usage, modeled estimates from the GBD can be a useful tool for quantifying severity, assessing international health disparities, comparing the disability posed by hearing loss to disparate diseases, and making policy or resource allocation decisions. The GBD study links estimates of hearing loss prevalence and severity to disability weights, a quantitative measure of health loss posed by a condition. This allows researchers and policymakers to assess the impact of hearing loss across different conditions, ages, and locations (Olusanya et al. 2019). Moreover, GBD estimates link hearing aid prevalence to estimates of hearing aid use, tinnitus, and occupational noise exposure, providing researchers, clinicians, and policymakers a systematic analysis of many of the clinically relevant aspects of hearing loss treatment and prevention. This article introduces key methods used to estimate global hearing loss from 1990 to 2019, key results from the United States, limitations in hearing loss methods, and areas of future development.

## MATERIALS AND METHODS

Estimates presented in this article are sourced from the Global Burden of Disease (GBD) study, which produces modeled estimates of global hearing loss prevalence and hearing aid use. The GBD study is a systematic analysis of the over 300 diseases, injuries, and risk factors in 204 countries and territories; details are described elsewhere (Vos et al. 2020). While the GBD study produces global estimates of many conditions, this article specifically highlights hearing loss results from the United States. Hearing loss and hearing aid use modeling for the GBD has been described in detail elsewhere and will be summarized later (“Hearing Loss Prevalence and Years Lived with Disability, 1990–2019: Findings from the Global Burden of Disease Study 2019—The Lancet” 2021; Orji et al. 2020). This study complies with the Guidelines for Accurate and Transparent Health Estimates Reporting recommendations (Stevens et al. 2016). Estimates are made publicly available via GBD Compare (https://vizhub.healthdata.org/gbd-compare/).

### Health Metrics in Hearing Loss Estimation

Prevalence is defined as the number of individuals who currently have a disease or condition, expressed either as a count or divided by the general population (provided as a percentage, where the denominator is 100 individuals). In addition to prevalence, the GBD utilizes a custom measure to estimate the impact of a nonfatal condition: years lived with disability (YLDs). YLDs quantify the amount of disability associated with a condition and are calculated by multiplying the severity-specific prevalence of a condition by its associated disability weight. Disability weights range from 0 (meaning perfect health) to 1 (which is equivalent to death). Weights were derived from a series of representative multinational surveys that made pair-wise comparisons of the severity of different health states (Salomon et al. 2015). Probit regressions were run on pooled data to translate the relative severity of each health state into disability weight values. YLDs enable quantitative comparisons of the burden of different conditions by accounting for both prevalence and the impact of disease severity on quality of life.

### Definition of Hearing Loss and Tinnitus

We defined hearing loss as measured by the softest sound an individual could hear in their better ear, taken as the pure-tone average of frequencies at 0.5, 1, 2, and 4 kHz. This definition aligns with the World Health Organization definition of hearing loss. We report the prevalence of hearing loss by six mutually exclusive severities (Table 1) based on decibel (dB) ranges: mild hearing loss (20 to 34 dB), moderate (35 to 49 dB), moderately severe (50 to 64 dB), severe (65 to 79 dB), profound (80 to 94 dB), and complete hearing loss (>95 dB) (“Hearing Loss Prevalence and Years Lived with Disability, 1990–2019: Findings from the Global Burden of Disease Study 2019—The Lancet” 2021).

**TABLE 1.** GBD severity thresholds and disability weights, with 95% uncertainty intervals

Health State	Lay Description	Range (dB)	Disability Weight (95% UI)
Normal	N/A	0–19	0
Mild	Has great difficulty hearing and understanding another person talking in a noisy place (for example, on an urban street).	20–34	0.01 (0.004–0.019)
Mild with ringing	Has great difficulty hearing and understanding another person talking in a noisy place (for example, on an urban street), and sometimes has annoying ringing in the ears.		0.021 (0.012–0.036)
Moderate	Is unable to hear and understand another person talking in a noisy place (for example, on an urban street), and has difficulty hearing another person talking even in a quiet place or on the phone.	35–49	0.027 (0.015–0.042)
Moderate with ringing	Is unable to hear and understand another person talking in a noisy place (for example, on an urban street), and has difficulty hearing another person talking even in a quiet place or on the phone, and has annoying ringing in the ears for more than 5 minutes at a time, almost every day.		0.074 (0.049–0.107)
Moderately severe	[Health state created from moderate and severe categories as recommended by expert collaborators.]	50–64	0.092 (0.064–0.129)
Moderately severe with ringing	[Health state created from moderate and severe categories as recommended by expert collaborators.]		0.167 (0.115–0.231)
Severe	Is unable to hear and understand another person talking, even in a quiet place, and unable to take part in a phone conversation. Difficulties with communicating and relating to others cause emotional impact at times (for example worry or depression).	65–79	0.158 (0.105–0.227)
Severe with ringing	Is unable to hear and understand another person talking, even in a quiet place, and unable to take part in a phone conversation, and has annoying ringing in the ears for more than 5 minutes at a time, almost every day. Difficulties with communicating and relating to others cause emotional impact at times (for example worry or depression).		0.261 (0.175–0.360)
Profound	Is unable to hear and understand another person talking, even in a quiet place, is unable to take part in a phone conversation, and has great difficulty hearing anything in any other situation. Difficulties with communicating and relating to others often cause worry, depression, and loneliness.	80–94	0.204 (0.134–0.288)
Profound with ringing	Is unable to hear and understand another person talking, even in a quiet place, is unable to take part in a phone conversation, has great difficulty hearing anything in any other situation, and has annoying ringing in the ears for more than 5 minutes at a time, several times a day. Difficulties with communicating and relating to others often cause worry, depression, or loneliness.		0.277 (0.182–0.387)
Complete	Cannot hear at all in any situation, including even the loudest sounds, and cannot communicate verbally or use a phone. Difficulties with communicating and relating to others often cause worry, depression or loneliness.	95+	0.215 (0.144–0.307)
Complete with ringing	Cannot hear at all in any situation, including even the loudest sounds, and cannot communicate verbally or use a phone, and has very annoying ringing in the ears for more than half of the day. Difficulties with communicating and relating to others often cause worry, depression or loneliness.		0.316 (0.212–0.435)

Hearing loss is also reported by the dimension of tinnitus status, which is characterized by being bothered by ringing, roaring, or buzzing in the ears lasting 5 min or more. This definition was obtained from U.S. National Health and Nutrition Examination (NHANES) surveys, which report the proportion of hearing-impaired respondents who self-report tinnitus (“NHANES 2009 to 2010: Audiometry Data Documentation, Codebook, and Frequencies”). We utilized the following NHANES questions from the audiometry questionnaire to classify tinnitus: “In the past 12 mo, {have you/has SP} ever had ringing, roaring, or buzzing in {your/his/her} ears?” and “How often did this happen? Would you say...” The possible responses to the latter question were “Almost always,” “At least once a day,” “at least once a week,” “at least once a month,” and “less frequently than once a month.” Individuals with mild hearing loss who currently experienced ringing at least once a month were considered tinnitus cases. Individuals with moderate to severe hearing loss who experienced ringing, roaring, or buzzing at least once a day were considered tinnitus cases. Additionally, individuals with complete hearing loss who reported that they almost always had ringing were considered tinnitus cases. The definition of tinnitus varies by severity to closely map to the GBD lay descriptions for hearing loss concurrent with tinnitus, which were derived by the GBD Hearing Loss Expert Group (Table 1).

Each severity of hearing loss is associated with a corresponding disability weight, used to reflect the relative impact of this health state on functional health. Disability weights increase with severity and tinnitus co-occurrence (Table 1). For example, a case of severe hearing loss with ringing has a disability weight of 0.267, which is less than a case of terminal liver cancer (DW 0.451) but greater than a case of near vision loss (DW 0.011) (Measuring the Global Burden of Disease | NEJM; Salomon et al. 2015). Following the completion of disability weight surveys, the moderately severe hearing loss categories were created so the threshold for each severity category was 15 dB away from the threshold for the following severity category, reflecting the minimum variation in thresholds considered clinically relevant (Olusanya et al. 2019). The disability weights for these categories were calculated by averaging the disability weights of the categories immediately above and below.

### Input Data

To estimate the burden of hearing loss globally, we utilized survey data obtained through systematic review of published epidemiologic studies. Additionally, we deliberately include microdata sources and sources referred to IHME through collaborator input, such as NHANES surveys. Nonrepresentative surveys, surveys that did not report bilateral hearing loss, and surveys that did not employ pure-tone audiometry as a diagnostic tool were excluded. We used NHANES individual-level data to adjust data that did not report hearing loss prevalence by the reference categories employed by the GBD. This was done by pairing data that reported prevalence according to different severity categories, then running a model on the estimated logit difference between prevalence of the reference severity category and alternative severity category. Members of the core IHME research team for this topic area had full access to the underlying data used to generate estimates presented in this paper. All other authors had access to, and reviewed, estimates as part of the GBD study and research evaluation process, which includes additional stages of internal IHME and external formal collaborator review. Input data are publicly available through the Global Health Data Exchange (ghdx.healthdata.org).

The key data source utilized to estimate hearing loss, hearing aid use, and tinnitus concurrence in the United States was the NHANES (National Health and Nutrition Examination) Surveys for the years 1999 to 2000, 2001 to 2002, 2003 to 2004, 2005 to 2006, 2007 to 2008, and 2009 to 2010 (“NHANES Survey Methods and Analytic Guidelines”). NHANES audiometric data covers individuals aged 20 to 69 from 1999 to 2004, aged 12 to 19 in 2007 to 2008, and people aged 12 to 19 and 70+ in 2005 to 2006 and 2009 to 2010. Hearing aid use and tinnitus concurrence data were additionally included for NHANES years 2011 to 2012. NHANES is a nationally representative, multiyear survey of the health status of adults and children in the United States, combining self-reported information with physical examinations (“NHANES—About the National Health and Nutrition Examination Survey” 2020).

We utilized the following NHANES question to assess hearing aid use: “{Have you/Has SP} ever worn a hearing aid?” In addition to NHANES data, we utilized hearing aid use data from the Norway Nord-Trøndelag study (Engdahl et al. 2005) and U.K. survey data (A. Davis et al. 2007) on tinnitus concurrence. We similarly utilized International Labour Organization data on occupational noise exposure and the relative risk of hearing loss among those exposed to occupational noise to estimate hearing loss attributable to occupational noise exposure (described elsewhere (“Hearing Loss Prevalence and Years Lived with Disability, 1990–2019: Findings from the Global Burden of Disease Study 2019—The Lancet” 2021).

### Modeling Strategy

To model hearing loss, we ran severity-specific models of hearing loss using a Bayesian meta-regression tool called DisMod-MR 2.1 (Vos et al. 2020). DisMod-MR 2.1 is a hierarchical Bayesian meta-regression tool, which enables us to leverage information across age, sex, location, and time to inform estimates in data-sparse ages, sexes, locations, and years. These models estimated mild, moderate, severe, moderately severe, profound, complete, and no hearing loss by year, age, sex, and location. We additionally ran two proportion models: one model for hearing aid use (the proportion of hearing-impaired people who used a hearing aid), and one for the proportion of hearing-impaired people who had age-related or other hearing loss. DisMod-MR 2.1 models primarily derived uncertainty from the uncertainty recorded in input data. Measurement uncertainty was established based on heterogeneity in prevalence estimates in NHANES studies 1988 to 2010. Uncertainty of model estimates was estimated by calculating the 25th and 975th ordered value from 1000 posterior runs of each model.

We scaled severity-specific models of hearing loss prevalence (ranging from no hearing loss to complete) to sum to the entire population in a series of proportional scaling adjustments. We then adjusted global, national, and state-level prevalence estimates downward based on hearing aid use, assuming that individuals who use a hearing aid have better hearing acuity than those who do not. First, we calculated hearing aid use for each severity of hearing loss using NHANES and Nord-Trøndelag data by running a logistic regression on age, severity, and sex:


$$\begin{array}{l} logit\left(use\right)=\beta age+\beta severity+\beta sex \end{array}$$

We then calculated severity-specific hearing aid use for all by scaling regression outputs based on estimates of total hearing aid use from DisMod-MR 2.1:


$$\begin{array}{l} use_{location,severity}=use_{NorwayandUS,severity}*\frac{use_{location,allseverities}}{use_{Norway,allseverities}} \end{array}$$

To adjust estimates of hearing loss prevalence for hearing aid use, we shifted the proportion of individuals who used a hearing aid in each location down one severity level of hearing loss. We conducted this adjustment due to an absence of data on the improvement in hearing acuity produced by hearing aid usage. We assumed that all individuals who use a hearing aid use one regularly. We assumed no improvement in hearing acuity from hearing aid use for individuals with complete hearing loss, under the assumption that amplification devices hold little utility on individuals with extensive cochlear or sensorineural damage (Lesica 2018).

We additionally split estimates of hearing loss by four etiologies: congenital abnormalities, chronic otitis media, meningitis, and age-related and other factors. We maintained uniform disability weights for hearing loss across etiology. We assumed that all hearing loss occurring at birth was congenital, and estimated hearing loss due to chronic otitis media and meningitis based on models of total prevalence of chronic otitis media and meningitis (described elsewhere) (Vos et al. 2020). For ages under 20, we proportionally scaled estimates of each cause of hearing loss so they summed to the prevalence of total hearing loss. After age 20, age-related and other hearing loss was treated as the residual between the sum of other causes of hearing loss and estimates of total hearing loss.

Finally, we estimated the proportion of hearing-impaired people with tinnitus using data from NHANES and United Kingdom (A. Davis et al. 2007) surveys. We utilized data from NHANES and UK surveys on the proportion of hearing-impaired people with tinnitus at each severity level, pooling this data, and generating uncertainty from a beta distribution. We applied the proportion of people with tinnitus at each severity of hearing loss to etiology-specific estimates of hearing loss, assuming the same distribution of tinnitus across all causes of hearing loss. This process produced final estimates of hearing loss prevalence by age, sex, location, severity, etiology, and tinnitus status. We calculated YLDs by multiplying prevalence estimates by their corresponding disability weight values, incorporating uncertainty from disability weight estimation and underlying prevalence values. We age-standardized estimates by multiplying age-specific prevalence by age weights for a standard reference population to account for population structure differences between countries.

### Occupational Noise Exposure

Occupational noise exposure is a risk factor for age-related and other hearing loss in the GBD study. Occupational noise is defined as the proportion of the population exposed to 85 + dB of noise. Data from the International Labour Organization were used to model occupational noise exposure by year, age, sex, and location. We sourced input data on the population prevalence of hearing loss due to occupational noise exposure from published studies from Australia and the United Kingdom (Agrawal et al. 2008; Davis 1989; Wilson et al. 1999). Data on the excess proportion of individuals with hearing loss due to occupational noise exposure was obtained by age, sex, and severity of exposure from Nelson et al. (2005). The relative risk of hearing loss due to occupational noise exposure was estimated as shown below:


$$\begin{array}{l} RR=1+\frac{excessprevalenceofhearingloss}{populationprevalenceofhearingloss} \end{array}$$

Modeling of hearing loss due to occupational noise exposure is described in greater detail elsewhere (GBD 2016 Occupational Risk Factors Collaborators 2020).

## RESULTS

### Hearing Loss in the United States

**Fig. 1.**
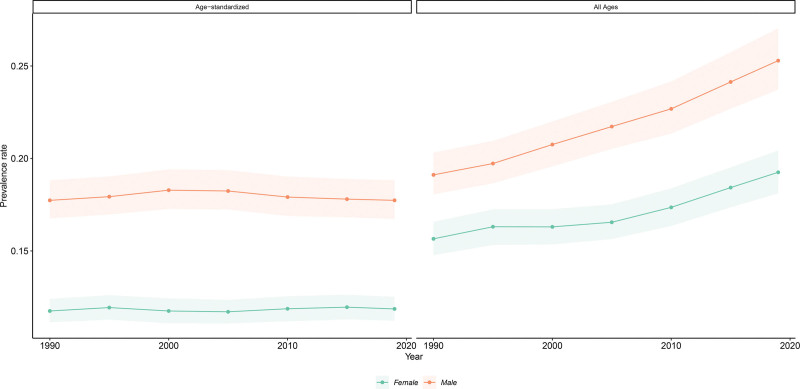
Age-standardized and all-ages hearing loss prevalence, 1990 to 2019, with 95% uncertainty intervals. Modeled data from the Global Burden of Disease study. Data were age-standardized according to the reference global population from the Global Burden of Disease study. All-ages prevalence calculated by summing prevalent cases across all age groups.

An estimated 72.88 million (68.53 to 77.30) people in the United States had hearing loss in 2019. This accounted for one in five people, or 22.2% (20.9 to 23.6) of the total population. Age-standardized prevalence was higher in males (17.7% [16.7 to 18.8]) compared with females (11.8% [11.2 to 12.5]) (Fig. 1). Hearing loss was the most prevalent sensory disorder in the United States and the sixth leading cause of YLDs after major depression, opioid use disorders, type 2 diabetes, other musculoskeletal disorders, and low back pain.

**Fig. 2.**
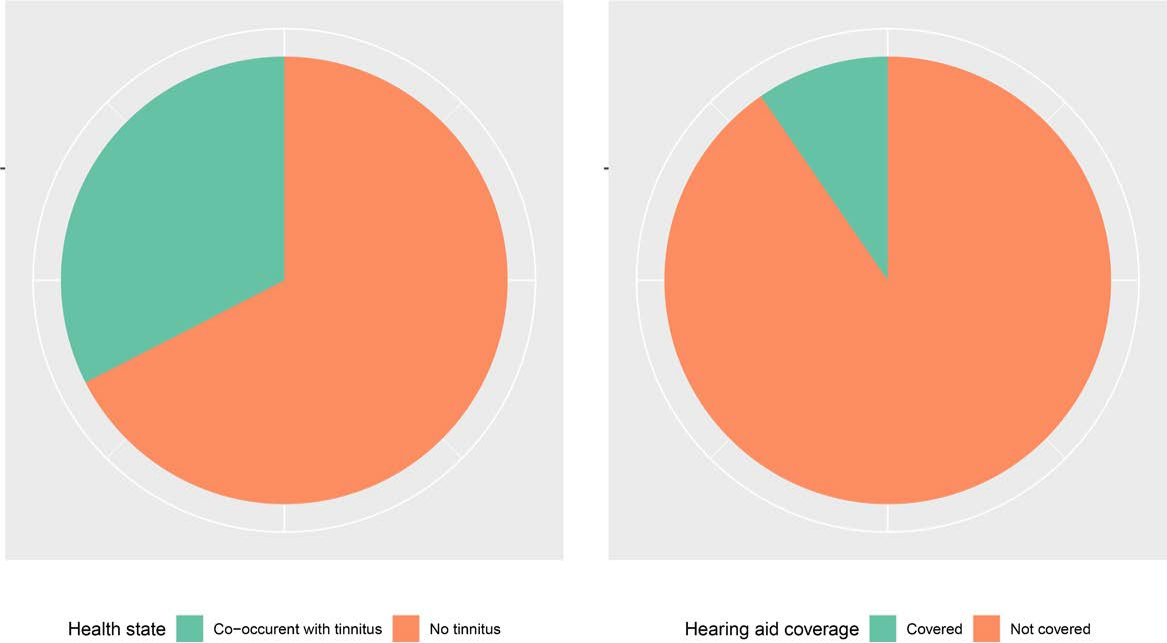
(A) Hearing loss prevalence by tinnitus co-occurrence, (B) Hearing aid use for individuals with mild to profound hearing loss, 2019, all ages. Modeled data from the Global Burden of Disease study.

Relative to other countries, the United States was ranked 148th of 204 countries in global rankings of age-standardized hearing loss prevalence but third in absolute number of cases due to large population size, after China and India. Of the 71.92 million (67.71 to 76.31) Americans with hearing loss that was mild to profound in severity, an estimated 6.97 million (6.11 to 8.01) utilized a hearing aid (9.7% [95% UI 8.1 to 11.0]). Moreover, an estimated 32.5% (95% UI 31.9 to 33.2) of Americans experienced hearing loss concurrent with tinnitus (Fig. 2).

### Hearing Loss by Severity

In 2019, the majority of hearing losses were of moderate or mild severity (Table 2). Moderate hearing loss accounted for 25.0% (22.4 to 27.5) of cases (18.22 million people [15.85 to 20.62]), with an all-age prevalence rate of 5.6% (4.8 to 6.3). Mild hearing loss accounted for 64.3% (61.0 to 67.6) of total hearing loss cases (46.85 million [43.88 to 50.07]), with an all-age crude prevalence of 14.3% (13.4 to 15.3). The remaining hearing loss cases ranged from moderately severe hearing loss to complete hearing loss.

**TABLE 2.** Estimated hearing impairment prevalence (all-ages and age-standardized), prevalent cases, and YLDs by severity in 2019, with 95% uncertainty intervals

Severity	Prevalence, All Ages (%)	Prevalence, Age Standardized (%)	Prevalent Cases	YLDs
Mild hearing loss	14.3 (13.4–15.3)	9.9 (9.3–10.5)	46.85 million (43.88–50.07)	597,200 (310,300–1.04 million)
Moderate hearing loss	5.6 (4.8–6.3)	3.3 (2.9–3.8)	18.22 million (15.85 – 20.62)	622,400 (394,200–909,300)
Moderately severe hearing loss	1.5 (1.3–1.8)	0.9 (0.8–1.1)	5.02 million (4.14–6.06)	467,100 (314,100–648,100)
Severe hearing loss	0.3 (0.2–0.4)	0.2 (0.2–0.2)	985,400 (764,700–1.26 million)	161,600 (104,800–230,200)
Profound hearing loss	0.3 (0.2–0.3)	0.2 (0.1–0.2)	837,600 (652,500–1.07 million)	173,500 (113,000–252,100)
Complete hearing loss	0.3 (0.2–0.4)	0.2 (0.2–0.2)	962,500 (775,500–1.18 million)	215,500 (139,700–309,600)

**Fig. 3.**
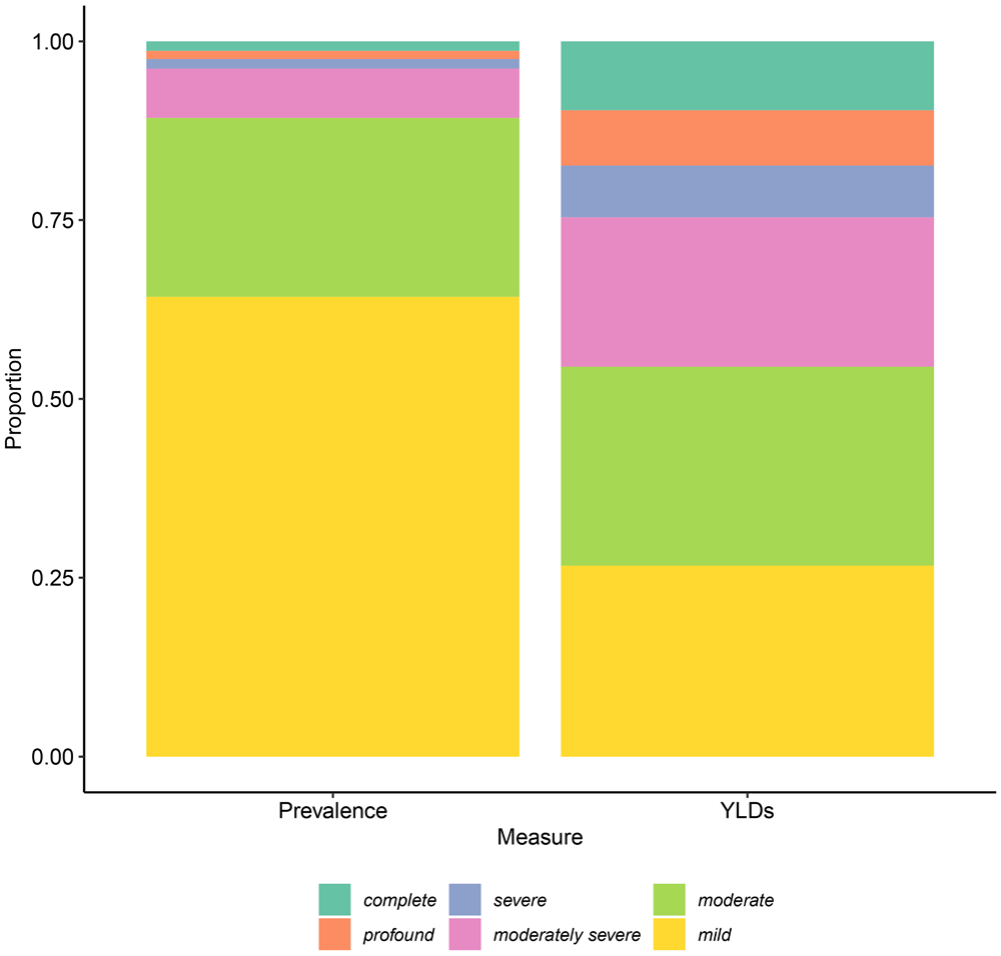
Proportion of hearing loss cases and YLDs by severity, 2019, all ages. Modeled data from the Global Burden of Disease study. All-ages prevalence calculated by summing prevalent cases across all age groups. YLDs, years lived with disability.

Of the 2.24 million (1.56 to 3.11) YLDs produced by hearing loss in the United States in 2019, 23.0% (13.4 to 34.7) were attributable to mild cases and 25.0% (19.7 to 30.7), 24.1% (18.0 to 30.4), and 8.1% (5.4 to 11.1) were attributable to moderate, moderately severe, and severe cases, respectively (Fig. 3). Hearing loss accounted for 3.6% (2.8 to 4.7) of all U.S. YLDs in 2019; 11.2% (10.2 to 12.4) of age-related and other hearing loss YLDs were attributable to occupational noise exposure. Age-related and other hearing loss was the third leading cause of YLDs for Americans over age 70, preceded only by low back pain and chronic obstructive pulmonary disease.

**Fig. 4.**
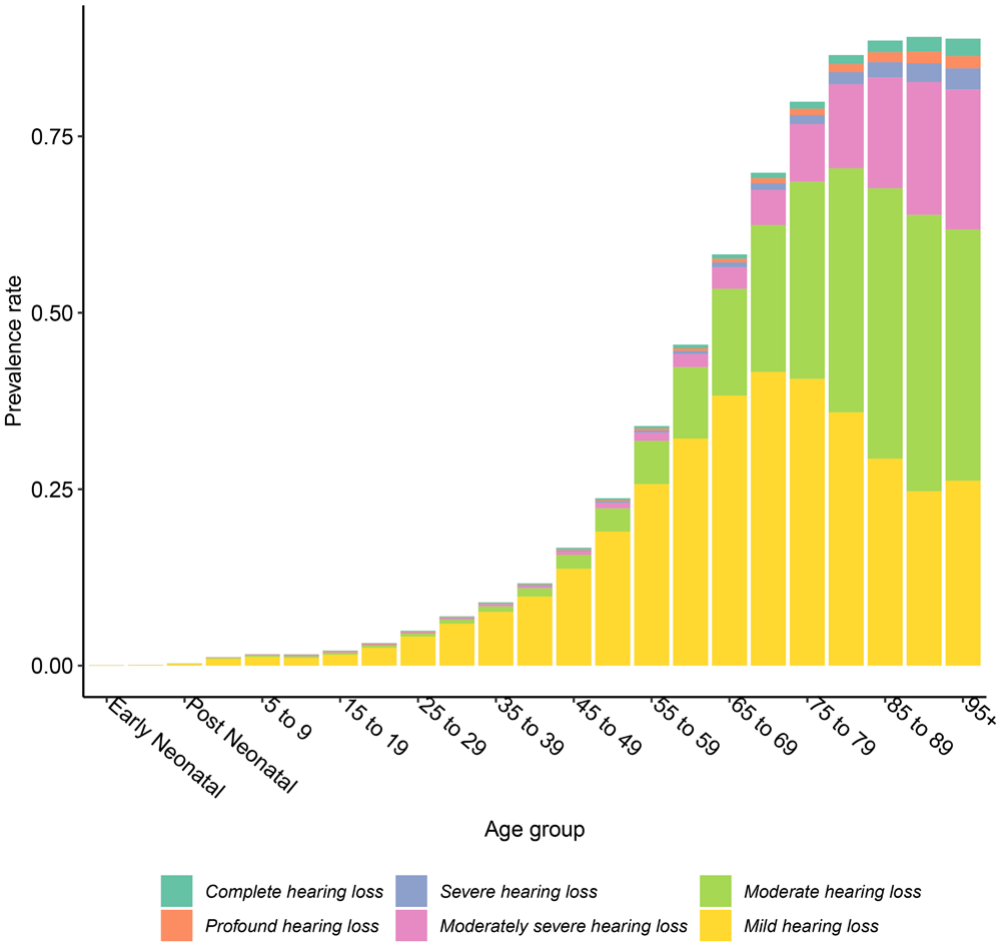
Hearing loss prevalence % by age and severity, 2019.* Modeled data from the Global Burden of Disease study. *Early neonatal = 0–6 days and postneonatal = 28–364 days.

Hearing loss prevalence and severity increased with age (Fig. 4). In 2019, 83.0% (81.5 to 84.3) of all Americans with hearing loss were over age 50, and 39.4% (35.7 to 43.0) of these people had hearing loss that was moderate or more severe. In contrast, 17.8% (14.4 to 20.8) of people with hearing loss under 50 had a case that was moderate or more severe.

### Temporal Trends

The all-age prevalence of hearing loss in the United States increased from 17.3% (16.4 to 18.3) in 1990 to 22.2% (20.9 to 23.6) in 2019, a 28.1% (25.7 to 30.8) increase. This difference was statistically significant (4.9%, 95% UI 4.4 to 5.4). Moreover, the number of hearing loss cases increased from 43.99 million (41.61 to 46.49) in 1990 to 72.88 million (68.53 to 77.30) in 2019, an increase of 65.7% (62.6 to 69.2). Much of this increase was driven by mild cases, which increased 64.4% (60.5 to 68.7) from 28.50 million (26.84 to 30.22) in 1990 to 46.85 (43.88 to 50.07) in 2019. In contrast to all-age prevalence, age-standardized prevalence remained constant, at 14.6% (13.8 to 15.3) of the population in 1990 and 14.7% (13.9 to 15.5) in 2019 (Fig. 1).

## DISCUSSION

Temporal trends between 1990 and 2019 demonstrate that hearing loss has become a growing source of disability in the United States. As the leading sensory disorder and the third leading cause of disability in Americans over 70, hearing impairment significantly impacts the health of older adults. While many cases of hearing loss in 2019 were mild, disability was concentrated in more severe cases, which should be treated through hearing aids, assistive technologies, and other forms of hearing healthcare (“WHO | *World Report on Hearing*”). Moreover, increases in all-age hearing loss prevalence complemented by stable age-standardized prevalence suggest that the increasing burden of hearing loss is closely tied to an aging population, and this dynamic will likely continue given projected shifts to an older age structure through the end of the century (“Fertility, Mortality, Migration, and Population Scenarios for 195 Countries and Territories from 2017 to 2100: A Forecasting Analysis for the Global Burden of Disease Study—The Lancet”).

An estimated 6 million of the 26 million (9.7 [8.1 to 11.0]) Americans with mild to profound hearing loss utilized a hearing aid, indicating high unmet need for healthcare services in groups most likely to benefit from intervention (Orji et al. 2020). Other studies suggest that this need is highest in underrepresented groups, including Black, rural, and low-SES populations (Nieman et al. 2016; Chan et al. 2017; Reed et al. 2021). Given U.S. policy proposals aimed at introducing over-the-counter hearing aid access, reducing cost, and removing the exclusion of hearing aid use from Medicare (Commissioner 2021; Dingell 2021), further research is needed to assess if unmet need reduces in future years.

An objective of Healthy People 2030 is to increase the number of adults with hearing loss who use a hearing aid by 2 percentage points (24.4% in 2018 to 26.4%)—a goal which will require commitment at the federal level to make it easier to acquire a hearing aid. The Medicare Audiologist Access and Services Act introduced in the U.S. House and Senate includes direct access to audiologists without requiring an order from a physician and streamlines coverage such that, if passed, audiologists will be able to provide the full range of diagnostic and treatment services within their scope of practice. Streamlining Medicare services is expected to save taxpayers $108 million over 10 years (Jong 2021). Though a step in the right direction to reducing barriers to care, unfortunately, coverage for hearing aids remains limited.

As the United States suffers from an audiologist shortage in many regions (Planey 2019), it is imperative that the American health system scales up workforce and health system capacity to meet this growing need. Public health approaches in the form of preventive and healthcare-based strategies are critical, including noise reduction programs, hearing aids, cochlear implants, reductions in ototoxic medication usage (such as diuretics and NSAIDs; Joo et al. 2020), and prevention of infectious diseases that can lead to hearing loss.

There are several strengths of our paper. First, our global modeling approach leveraged information on the severity, etiology, and distribution of hearing loss from varied analyses, included adjustment for hearing aid utilization, and incorporated severity-specific disability weights, which permit rigorous comparisons of disability between age groups, locations, time, and other causes of disease or injury.

While the GBD’s linkage between hearing loss audiometric thresholds, descriptions of functional health, and quantitative disability weights permit a systematic view of health loss produced by hearing impairment, it is important to note that these classifications may not capture the full range of functional health outcomes for each individual living with hearing loss. As audiometric measurements do not map directly onto real-world hearing acuity for every individual, depending on the type of hearing loss, even individuals with “normal” audiometric thresholds may experience functional limitations (Olusanya et al. 2019). Conversely, some individuals with milder hearing losses may experience negligible health loss, despite this classification. Moreover, disability weights were derived from comparative judgements made by nationally representative samples and not necessarily from individuals who experience hearing loss. Quantitative measures of disability produced by the GBD should be complemented by holistic measures of functional health, including self-reported measures from those impacted by hearing loss. There could be measurement errors in our estimates of hearing loss prevalence due to audiometric thresholds being collected in noisy environments during large population-based surveys. However, our main data source, NHANES, used a sound-isolating room and monitored ambient noise levels that met or exceeded industry standards (ANSI S3.1 1991). Per NHANES audiometric protocol, pure-tone audiometric testing was not conducted in environments with excessive noise levels (“NHANES 2009 to 2010: Audiometry Data Documentation, Codebook, and Frequencies”). Therefore, audiometric threshold measurement errors due to ambient noise should be minimal.

In this study, we were interested in reporting the burden of hearing loss among the US population. Considering tinnitus is often comorbid with hearing loss, and tinnitus is associated with increased mental distress and decreased quality of life (Nondahl et al. 2007; Reavis 2020), disability weights for hearing loss with and without tinnitus were used to estimate the number of years lived with hearing disability. We followed NHANES guidelines and define a tinnitus case as bothersome ringing, roaring, buzzing, or other sound present for at least 5 minutes. For some individuals, the occurrence of tinnitus and its adverse sequela may not manifest until tinnitus has occurred with some regularity. Therefore, GBD disability weights are only for hearing loss and hearing loss with frequently occurring tinnitus. A tinnitus case was defined as being present >5 min and had to occur at least monthly when paired with mild hearing loss, at least daily when paired with moderate to severe hearing loss, and constantly when paired with profound hearing loss. Consequently, for moderate or greater hearing losses, weekly, or monthly experiences of tinnitus were not counted as tinnitus cases. These case definitions of hearing loss with tinnitus do not capture all instances of tinnitus in the general population—suggesting that the true burden of hearing loss in the US population might be higher than reported.

A key limitation of our modeling approach was data sparsity, both globally and in the United States. Our models of hearing loss prevalence in the United States were primarily informed by NHANES surveys, which provide age-, sex-, and severity-specific information on loss, as well as data on the proportion of survey participants who utilized a hearing aid or experienced tinnitus. While NHANES surveys were valuable sources of information on hearing loss in the United States, NHANES data in our models were not reported at the state level, which reduces our capacity to make inferences about hearing loss burden by state. Moreover, due to the cyclic nature of data-seeking in the GBD study, NHANES data from 2011 to 2012 and 2015 to 2016 were not included in GBD 2019 estimates. These rounds of data were included in GBD 2020 estimates, and future rounds of NHANES surveys will be readily incorporated in future iterations of the GBD study. Although our modeling methods allow estimates to borrow strength from other high-income countries with more recent data, this impacts the ability to accurately estimate the burden of hearing loss in the United States over time, particularly in years after 2010. In conjunction with modeling methods that leverage temporal data from other locations in regions where new data are not available, this lack of newer data may mask temporal trends in NHANES data which suggest decreases in age- and sex-specific hearing loss prevalence over time (Hoffman et al. 2017). Our models also suffer from a lack of predictive covariates for hearing loss prevalence, which could otherwise be used to strengthen estimates in data-sparse locations. Estimates reported in this manuscript also are not disaggregated by race/ethnicity, although there is evidence that hearing loss prevalence varies by race (Agrawal et al. 2008).

While NHANES data provided audiological information on individuals over 5 years old, it did not include similar information for young children or infants. There was a lack of specific information on bilateral hearing loss in children in the United States, making it particularly difficult to produce accurate estimates of hearing impairment prevalence in younger ages. Sources such as the CDC Early Hearing and Detection program provide comprehensive estimates of hearing loss in American infants, but report information by ear instead of by person, and therefore were not included in our modeling approach.

Moreover, our estimates of hearing loss burden assume reduced burden of hearing loss in individuals who use a hearing aid, in the absence of data on the decibel-level improvement in hearing acuity produced by hearing aid usage. Our adjustment methods may be crude in nature, neglecting other components of hearing aid usage that may impact hearing acuity, including hearing aid fit, daily hours of use, technology style, and upkeep.

Due to insufficient information at the country level, a major limitation of the occupational noise methodology is that industry and occupation are primarily used as proxies for exposure, and we only included limited information on noise exposure levels within different industries. Further, use of hearing protection was only considered to a limited degree and with rather general assumptions regarding differences in exposures between low/middle-income and high-income countries. Additional data gaps included state-level data, information on the usage of hearing devices such as cochlear implants and other technologies, and information on the etiology of hearing impairment, which are not commonly assessed in surveys of hearing loss prevalence. More data in these areas are needed to rigorously assess the burden of hearing impairment in the United States, providing relevant information for domestic policymakers and hearing healthcare providers.

Hearing impairment is a growing problem that has detrimental impacts on language development, communication, and social wellbeing. Estimates indicate that hearing impairment is a leading source of disability in the United States. Despite high burden, hearing aid use is low, indicating unmet need for healthcare services that will grow in coming years if not complemented by health system scale-up. As populations age and prevalence rises, new data sources on the severity, etiology, and prevalence of hearing loss are needed to strengthen national estimates and target healthcare services toward key populations.

## ACKNOWLEDGMENTS

L.M.H. and P.S.B. report personal fees from the World Health Organization from a contract to conduct an analysis for the World Hearing report, paid directly to them. K.M.R. reports grants or contracts from the US Department of Defense (DoD JWMRP #160036, DoD JWMPR JW210396), and US Veterans’ Affairs (VA RR&D RX003888-01, VA RR&D C3701R), payments made to institution to support salary, and from US National Institutes of Health (NIH-NIDCD 1R13DC020098-01), outside the submitted work. T.W.B. reports grants or contracts from the European Union (Horizon 2020 and EIT Health), German Research Foundation (DFG), US National Institutes of Health, German Ministry of Education and Research, Alexander von Humboldt Foundation, Else-Kröner-Fresenius-Foundation, Wellcome Trust, Bill & Melinda Gates Foundation, KfW, UNAIDS, WHO, consulting fees from kfW on the OSCAR initiative in Vietnam, participation on a Data Safety Monitoring Board or Advisory Board with NIH-funded study “Healthy Options” (PIs: Smith Fawzi, Kaaya), Chair, Data Safety and Monitoring Board (DSMB), German National Committee on the “Future of Public Health Research and Education,” Chair of the scientific advisory board to the EDCTP Evaluation, Member of the UNAIDS Evaluation Expert Advisory Committee, National Institutes of Health Study Section Member on Population and Public Health Approaches to HIV/AIDS (PPAH), US National Academies of Sciences, Engineering, and Medicine’s Committee for the “Evaluation of Human Resources for Health in the Republic of Rwanda under the President’s Emergency Plan for AIDS Relief (PEPFAR),” University of Pennsylvania (UPenn) Population Aging Research Center (PARC) External Advisory Board Member, and leadership or fiduciary role in board, society, committee or advocacy group, paid or unpaid as Cochair of the Global Health Hub Germany (which was initiated by the German Ministry of Health); outside the submitted work. I.M.K. reports supposed for the present manuscript from the Bill & Melinda Gates Foundation and the World Health Organization. A Singh reports consulting fees from Crealta/Horizon, Medisys, Fidia, Two labs, Adept Field Solutions, Clinical Care Options, ClearView Healthcare Partners, Putnam Associates, Focus Forward, Navigant Consulting, Spherix, MedIQ, UBM, Trio Health, Medscape, WebMD, and Practice Point Communications; and the National Institutes of Health and the American College of Rheumatology; payment or honoraria for lectures, presentations, speakers bureaus, manuscript writing, or educational events from Simply Speaking; support for attending meetings or travel, or both from OMERACT, an international organization that develops measures for clinical trials and receives arm’s length funding from 12 pharmaceutical companies, when traveling to OMERACT meetings; participation on a data safety monitoring board or advisory board as a member of the FDA Arthritis Advisory Committee; leadership or fiduciary role in other board, society, committee or advocacy group, paid or unpaid, with OMERACT as a member of the steering committee, with the Veterans Affairs Rheumatology Field Advisory Committee as a member, and with the UAB Cochrane Musculoskeletal Group Satellite Center on Network Meta-analysis as a director and editor; stock or stock options in TPT Global Tech, Vaxart Pharmaceuticals, Atyu Biopharma, Adaptimmune Therapeutics, GeoVax Labs, Pieris Pharmaceuticals, Enzolytics Inc., Seres Therapeutics, Tonix Pharmaceuticals, and Charlotte’s Web Holdings, and previously owned stock options in Amarin, Viking, and Moderna Pharmaceuticals; all outside the submitted work.
